# Apoptosis-Promoting Effects of Hematoporphyrin Monomethyl Ether-Sonodynamic Therapy (HMME-SDT) on Endometrial Cancer

**DOI:** 10.1371/journal.pone.0137980

**Published:** 2015-09-14

**Authors:** Haizhu Sun, Wenjie Ge, Xin Gao, Shaoshan Wang, Shijian Jiang, Ying Hu, Miao Yu, Shaoshan Hu

**Affiliations:** 1 Department of Neurosurgery, Affiliated Hospital 2, Harbin Medical University, Harbin, 150001, China; 2 State Key Laboratory of Urban Water Resource and Environment, School of Chemical Engineering and Technology, Harbin Institute of Technology, Harbin, 150001, China; 3 Department of Obstetrics and Gynecology, Affiliated Hospital 2, Harbin Medical University, Harbin, 150001, China; 4 School of Life Science and Technology, Harbin Institute of Technology, Harbin, 150001, China; University of Quebec at Trois-Rivieres, CANADA

## Abstract

**Objective:**

The aim of the present study was to examine the apoptosis-promoting effects and mechanisms of hematoporphyrin monomethyl ether (HMME)-sonodynamic therapy (SDT) on endometrial cancer cells *in vitro*.

**Methods:**

Endometrial cancer cell samples were divided into four groups: 1) untreated control group, 2) HMME group, 3) pure ultrasound group, and 4) HMME combined with ultrasound, i.e. SDT group. CCK-8 method was utilized to assess the inhibiting effect of SDT on the proliferation of endometrial cancer cells. Optical microscope and field emission transmission electron microscopy were used to characterize the morphology changes of the cancer cells induced by the treatments. Apoptosis rate, reactive oxygen species (ROS) and mitochondrial membrane potential (MMP) were examined by flow cytometer. Fluorescence intensity measured by laser scanning confocal microscopy was used to explore the variation of intracellular calcium ion (Ca^2+^) concentration. Apoptosis-related proteins involved in both intrinsic and extrinsic apoptosis signallings were analyzed by western blot.

**Results:**

SDT can effectively induce the apoptosis of endometrial cancer cells. Compared with ultrasound which is known as an effective anti-tumor method, SDT leads to a significant improvement on suppression of cell viability and induction of apoptosis, together with more remarkable modifications on the morphology and substructure in both ultrasound sensitive and resistant endometrial cancer cells. Further studies reveals that SDT promotes ROS production, induces loss of MMP and increases intracellular Ca^2+^ concentration more efficiently than HMME or ultrasound alone. SDT groups also show a rather high expression of apoptosis-promoting proteins, including Bax, Fas and Fas-L, and a significant low expression of apoptosis-suspending proteins including Bcl-2 and Survivin. Meanwhile, both cleaved caspse-3 and caspase-8 are dramatically enhanced in SDT groups. Multiple pathways has been proposed in the process, including the intrinsic activation by excessive ROS and overloaded Ca^2+^, silencing survivin gene, and the extrinsic pathway mediated by the death receptor.

**Conclusion:**

Given its considerable effectivity in both ultrasound sensitive and resistant cells, SDT may therefore be a promising therapeutic method for treating endometrial cancers.

## Introduction

Endometrial cancer is one of common gynecological malignancies, traditionally treated by surgery and supplemented by chemotherapy and radiotherapy, which unfortunately have a rather low sensitivity to early or metastatic stage of endometrial cancer, normally accompanying with significant side-effects [1–2]. So far, it is still an open issue how early endometrial cancer can be treated while preserving fertility. And there is no report in the literature on the organ retention for patients at an intermediate or advanced stage. Obviously, an efficient approach with low toxicity is paramount.

The inducement of apoptosis is preferred in cancer treatment [3]. It has been demonstrated that ultrasound can cause apoptosis of a big variety of cells, such as myeloid leukemia cells, lymphocytes and sarcoma cells [4–6]. However, the delayed killing effect [7] together with the inefficient efficacy on ultrasound-resistant tumor cells may largely limit its clinic applications. Recently, as a combination of ultrasound and sonosensitizers, sonodynamic therapy (SDT) has become a fascinating non-invasive anti-tumor method with high efficiency and high selectivity in the tumor cells and no obvious influence on adjacent normal tissues [8–10]. Among diverse chemical agents applied in SDT, hematoporphyrin monomethyl ether (HMME) is particular popular as a sonosensitizer due to its optimal optical properties and satisfactory preferential retention in tissue [11]. More importantly, HMME has a much higher uptake rate by tumor than normal tissues [10], and this high selectivity ensures the ultrasound energy to be focused specifically on the cancer cells.

The application of SDT on gynecologic malignancies is quickly emerging. Though a previous study *in vitro* did show that methylene blue-mediated SDT can significantly increase the reactive oxygen species in ovarian cancer cells and hence induce apoptosis [12], SDT effects on endometrial cancer remain unexplored up to now.

In this study, influence of HMME-SDT on both ultrasound sensitive and resistant endometrial cancer cells has been investigated *in vitro*. Apoptosis-promoting effects and mechanisms are discussed in the light of available information.

## Materials and Methods

### 2.1. Cell Culture

Human endometrial adenocarcinoma cell lines Ishikawa and HEC-1-a were purchased from Shanghai Bogoo Biotechnology. Co., Ltd. The cells were cultured in a RPMI1640 (Hyclone, Thermo) solution containing 10% fetal calf serum (Hyclone, Thermo) and incubated in a saturated humidity incubator (HF240, Heal Force) at 37°C with 5% CO_2_. The experiments were carried out when the cells reached the logarithmic growth phase.

### 2.2. Cell Survival Rate

Cells in the logarithmic growth phase were placed in 0.25% trypsin for digestion and centrifugation and prepared into a single-cell suspension with a concentration of 1×10^5^/ml. The cells were then seeded in 6-well plates. There were four sample groups as follows: 1) untreated control group (control), 2) HMME group (HMME), 3) pure ultrasound group (ultrasound), and 4) ultrasound combined HMME group (SDT). For SDT group, HMME was added one hour before the ultrasound interference, which was 1.0 W/cm^2^ at 1 MHz for 60 s for Ishikawa and 2.0 W/cm^2^ at 1 MHz treated 240 s for HEC-1-a. And the final concentration of HMME was 15 μg/ml for Ishikawa and 50 μg/ml for HEC-1-a. A wet sponge was placed between the bottom of culture plate and the ultrasound probe to prevent multiple reflections of ultrasound. After irradiation, the cells were digested and collected immediately, then transferred to 96-well plates after adjusting the concentration, with 100 μl in each well. Two hours later, 10 μl Cell Counting Kit-8 reagent (CCK-8, Dojindo) was added to each well. After one hour of further culture, absorbance at 490 nm was measured using an automatic enzyme mark instrument (Multiskan MK3, Thermo) [13].

### 2.3. Preparation for Characterization of the Cell Morphology and Substructure

The morphology and adherent growth of cells were observed 6h after the four different treatments by using an inverted microscope (Olympus CKX4). The cells were digested in 0.25% trypsin for 90s, washed by phosphate buffered saline, then centrifuged at 2000 r/min for 5min. Removing the supernatant, the cells were fixed between 2.5% glutaraldehyde and 1% osmic acid, dehydrated by ethanol and acetone, embedded in Epon812 epoxy resin, and cut by III ultra-thin slicer. The samples were then double-dyed with uranyl acetate and lead citrate, and then characterized using a field emission transmission electron microscopy (TEM, JEM-2010, JEOL).

### 2.4. Apoptosis Analysis

6h after the treatments, the cells of the four groups mentioned above were collected and counted, then preserved avoiding light for 15 min following an addition of 10 μl fluorescein isothiocyanate (FITC)-labeled AnnexinV (20μg/ml) for 1×10^6^ cells. Afterwards, 5 μl propidium iodide (PI) (50 μg/ml) was added. After reaction for 5min, 400 μl binding buffer was mixed. The specimens were tested by flow cytometer (BD FACSCanto) immediately.

### 2.5. Reactive Oxygen Species (ROS) and Mitochondrial Membrane Potential (MMP)

After ultrasonic irradiation, the cells were incubated for 2h. 2’7’-dichlorofluorescein diacetate (DCFH-DA, Beyotime) and Rhodamine123 (Sigma) with a final concentration of 10 μmol/L and 200 nmol/L were added to each sample for the ROS and MMP test. In both cases, the cells were cultured at 37°C with 5% CO_2_ avoiding light for 30min. Fluorescence intensity was then measured by a flow cytometer at an excitation and emission wavelength of 480 nm and 520 nm for ROS test, and 490 nm and 530 nm for MMP test, respectively.

### 2.6. Determination of Intracellular Calcium (Ca) Ion Concentration

Endometrial cancer cells of control and SDT group were washed three times by phosphate buffer solution, loaded by Fluo-3/AM (Bio-Rad) with a final concentration of 10 μmol/L at 37°C for 45 min, and then transferred to a special small groove. Fluorescence intensity was tested by a laser scanning confocal microscope (Meridian, Insight-PlusIQ) at an argon laser excitation of 488 nm. The free Ca^2+^ concentration in cells was calibrated with the equation: [*Ca* ^2+^] = *K*_*d*_[(*F* − *F*_min_)/(*F*_max_ − *F*)], where *K*_*d*_ is 400 nMol, *F* is the measured relative fluorescence value, *F*_*max*_ is the maximum fluorescence value after the addition of 10 μmol/L 4-Br-A23187 high Ca liquid (10 mmol/L K_2_CaEGTA, 10mmol/L K-mops and 100 mmol/L KCl, Bio-Rad Company), *F*_*min*_ is the minimum fluorescence value after the addition of 10 mmol/L 4-Br-A23187 Ca-free solution [14]. This is a relative simple and low-cost method normally applied for intracellular Ca^2+^ concentration.

### 2.7. The Expression of Apoptotic-Related Proteins

Apoptosis-related proteins were analyzed 24 h after each treatment by western blot. Briefly, cells were harvested after different treatments. Proteins were separated by the SDS-PAGE gel and then transferred to PVDF membranes, which were firstly blocked with 5% skimmed milk and then incubated with specific antibodies as indicated in the figures at 4°C overnight. The horseradish peroxidase-conjugated secondaries were added afterwards. The expression of proteins was visualized by enhanced chemiluminescence (ECL) system (Thermo Scientific Pierce).

### 2.8. Statistical Analysis

All experiments were reproduced three times independently in the different cells. The SPSS Version 13.0 for Windows (SPSS Inc., Chicago, Illinois) was used for data analysis. Data are presented as mean ±square deviation (SD). The date was analyzed using unpaired T-test. P<0.05 was considered to be statistically significant.

## Results

### 3.1. Effect of SDT on Survival Rate of the Endometrial Cancer Cells

**Fig 1:**
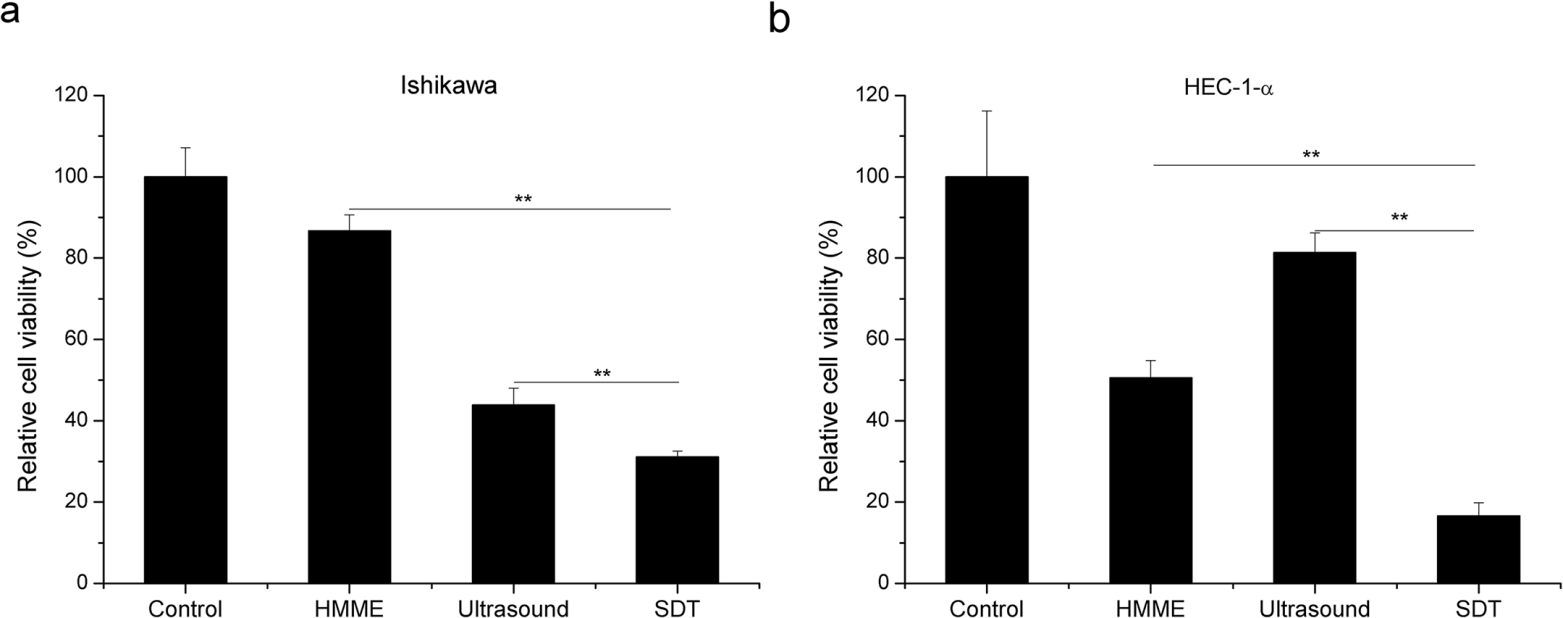
Effect of SDT on the survival rate of endometrial cancer cells, assessed by the CCK-8 method. CCK-8 assay was used to evaluate the cell viabilities in control, HMME, ultrasound and SDT groups in Ishikawa (a) and HEC-1-a (b) cells. HMME or ultrasound shows an obvious inhibitory effect on cell survival. SDT inhibits cell viability more effectively than either single treatment. Data are presented as the mean ± SD (n = 3), **P < 0.01.

In order to evaluate the effect of SDT on cell viability, a CKK-8 assay was applied to Ishikawa and HEC-1-a cells upon different treatments as indicated in Fig 1. The survival rate is decreased with single treatment of either HMME or ultrasound in both Ishikawa and HEC-1-a cells (Fig 1). Interestingly, cell viability of Ishikawa cells is decreased to 33.99 ± 4.06% with 1 MHz ultrasound (1.0 W/cm^2^) for 60 s; whilst no obvious growth inhibition is observed in HEC-1-a cells under the same condition (Refer to S1A Fig). Furthermore, it is found that the cell viability of HEC-1-a cells is maintained no less than 81.39 ± 4.83% upon treatment even with stronger ultrasound (2.0 W/cm^2^) for an extended duration from 60 s to 240 s (S1B Fig). This directly indicates the sensitivity of Ishikawa and resistance of HEC-1-a upon ultrasound treatment. Importantly, as shown in Fig 1, although ultrasound inhibits cell viability of endometrial cancer cells to different extend, the synergy of ultrasound with HMME can consistently and substantially arouse a largely enhanced killing effectivity, i.e. 165% and 115% as that of ultrasound alone in HEC-1-a and Ishikawa cells, respectively.

### 3.2. Effect of SDT on the Morphology and Substructure of the Endometrial Cancer Cells

**Fig 2:**
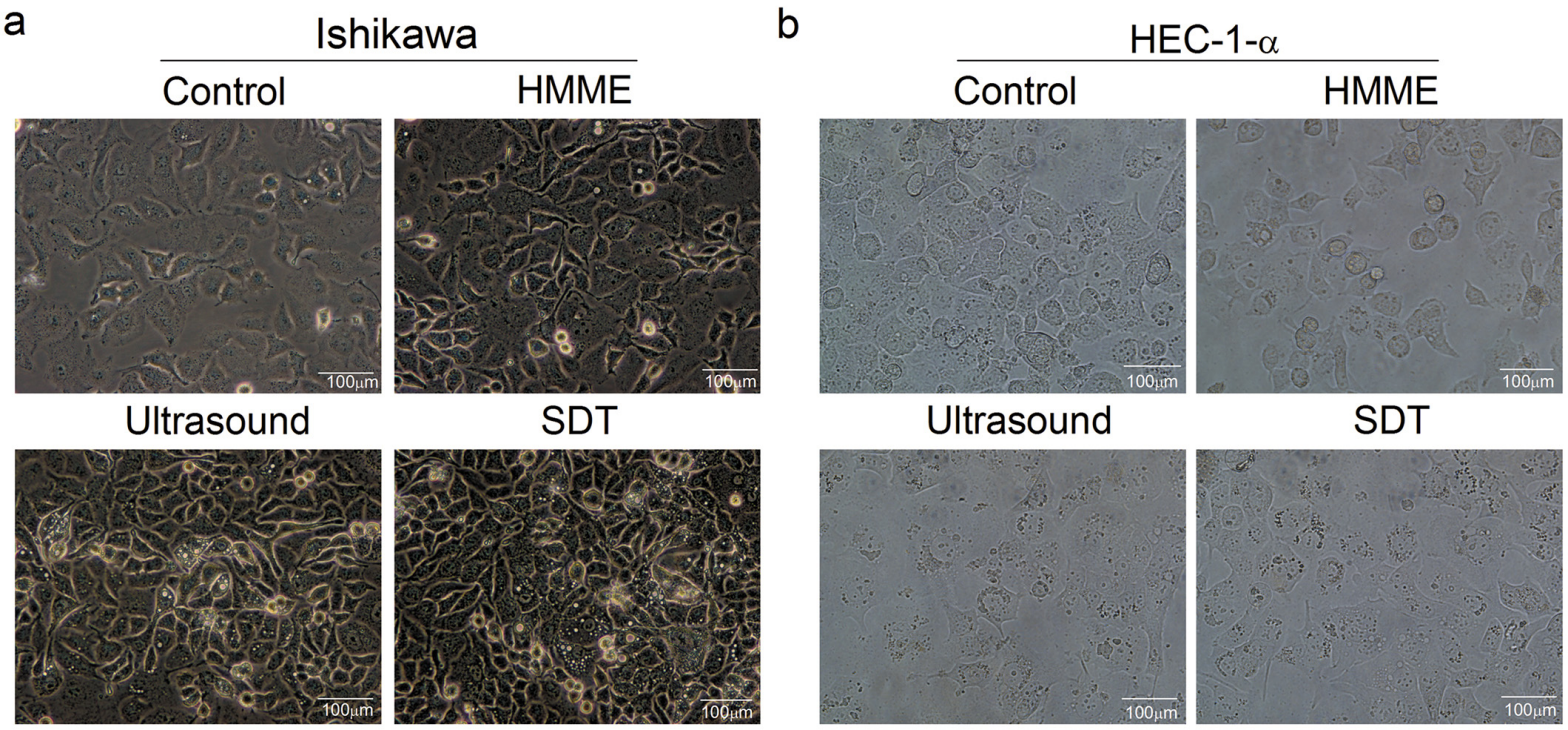
Effect of SDT on the morphology of the endometrial cancer cells. The morphology and adherent growth of Ishikawa (a) and HEC-1-a (b) cells were observed 6h after the four different treatments by using an inverted optical microscope (Olympus CKX4). Control groups show adherently grown cells with clear contour and good transparency; ultrasound and SDT group show poorly transparent cells, which are not firmly attached or even suspended. The magnification of all images is 40 times, scale bar = 100μm.

Seen from the images in Fig 2, for the control and HMME groups, most cells are grown adherently and uniformly into a ‘paving-stone’ structure, showing clear contour, good transparency and vitality; while cells of the ultrasound and SDT group are poorly transparent, not firmly attached or even suspended, and the morphology of part cells becomes circular.

**Fig 3:**
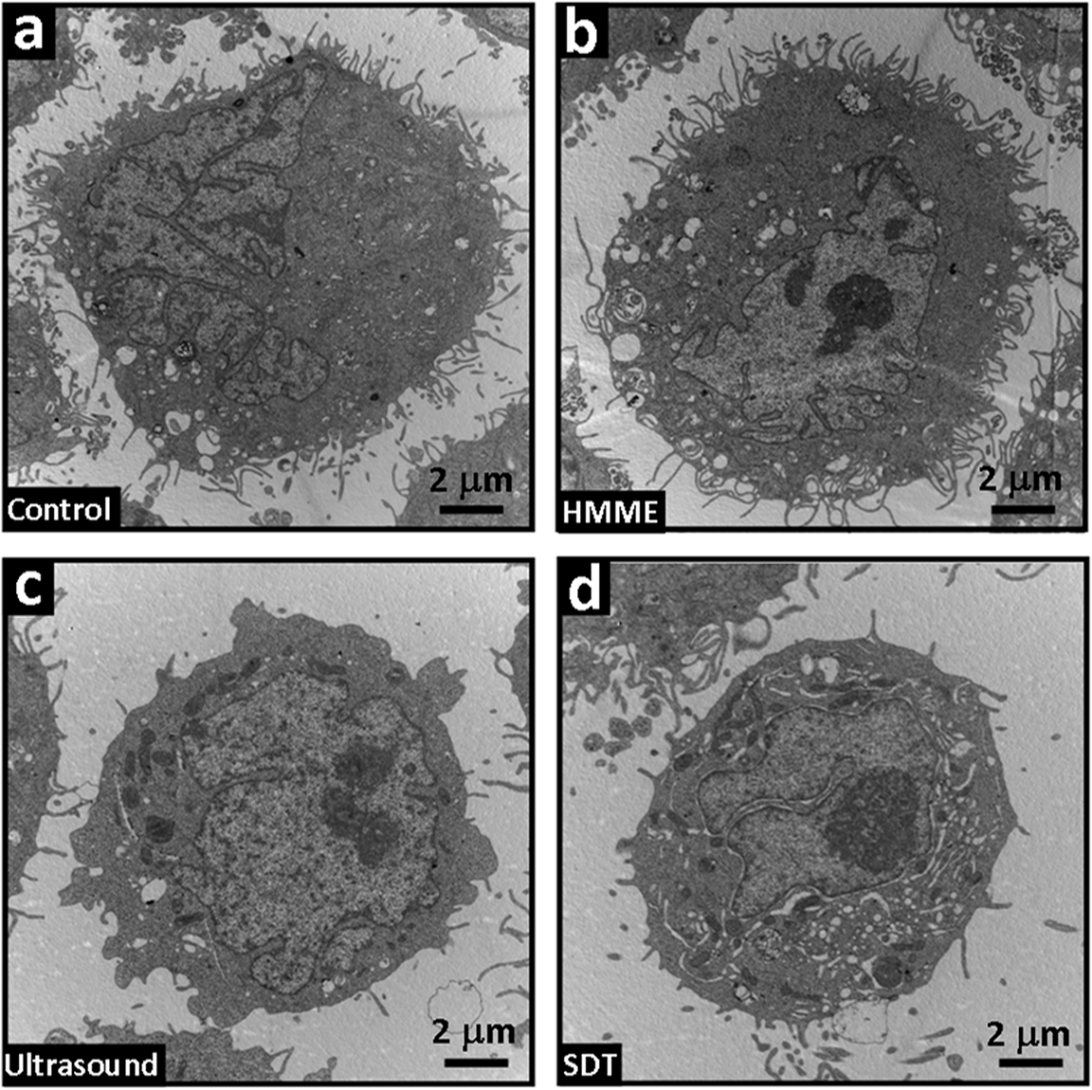
Effect of SDT on the substructure of the endometrial cancer cells. The substructure of the four group cells was characterized by transmission electron microscopy (TEM, JEM-2010, JEOL). (a) control and (b) HMME group, showing intact cell membrane and nuclear membrane, clear organelle structure and complete mitochondrial structure without obvious modification; (c) ultrasound group, showing tendency towards cell apoptosis, including coiled or congealed and marginalized cell nucleus chromatin, deformed and vacuolar mitochondria and increased lysosome; (d) SDT group, showing typical phenomena of apoptosis, where the nuclei are under obvious pyknosis into a heterogeneous block structure, with small apoptotic bodies, enlarged perinuclear gap and nuclear pore, expanded Golgi apparatus and endoplasmic reticulum under degranulation, as well as blurry or vanished mitochondrial cristae. The scale bar in all images is 2 μm.

More details can be explored from the TEM images in Fig 3. It is found that the cells of the control and HMME group possess intact cell and nuclear membranes, clear organelle structures and complete mitochondrial structures without obvious modification. With respect to Ultrasound group, the cell nucleus chromatin is obviously coiled or congealed and marginalized; the mitochondria become swollen, deformed and vacuolar, and the lysosome is increased, which are all indications towards cell apoptosis. For SDT group, typical phenomena of apoptosis can already be observed from the cells, where the nuclei are under obvious pyknosis into a heterogeneous block structure, showing small apoptotic bodies, enlarged perinuclear gaps and nuclear pores, expanded Golgi apparatus and endoplasmic reticulum under degranulation, as well as blurry or vanished mitochondrial cristae. Some cells even show accumulated glycogen granule in their cytoplasm with a decreased cell volume.

### 3.3. Effect of SDT on ROS Generation and MMP Reduction

**Fig 4:**
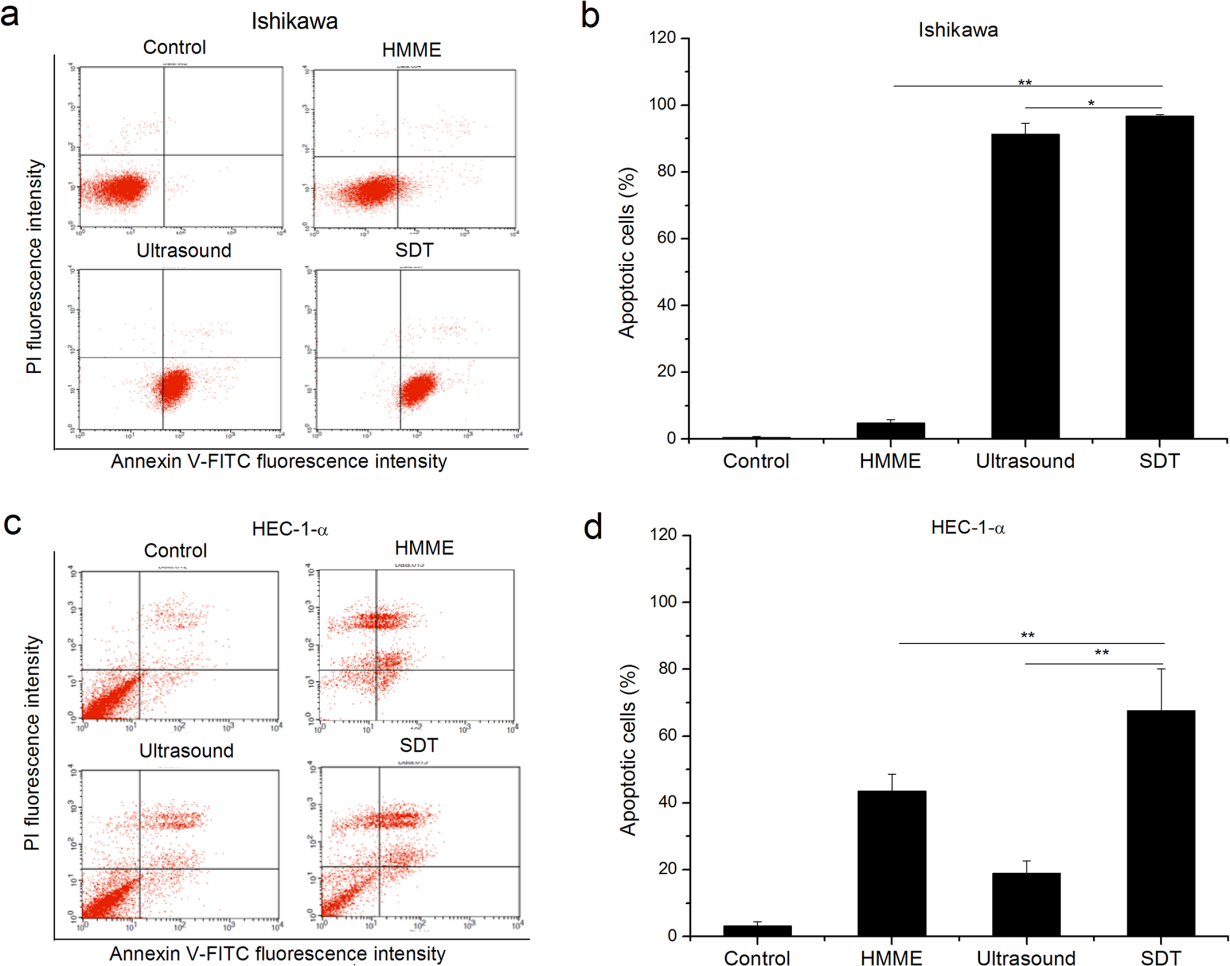
Analysis of apoptosis rate of endometrial cancer cells using flow cytometer. The apoptosis levels are much higher in SDT groups than the control, HMME or ultrasound groups in Ishikawa (a and b) and HEC-1-a (c and d) cells. Representative FACS profiles were shown in a and c. Histograms present mean ± SD of three independent experiments (b and d, * P < 0.05; ** P < 0.01.)

As shown in Fig 4, the apoptotic rate of SDT, ultrasound, HMME and the control group is 96.66±0.45%, 91.21±3.44%, 4.75±1.10% and 0.42±0.35%, respectively, in Ishikawa cells and 67.54±12.65%, 18.88±3.73%, 43.50±5.02% and 3.09±1.37%, respectively, in HEC-1-a cells. The induced apoptosis is inversely correlated with the cell viability determined by CCK-8 assay in the four different groups (S1 Fig and Fig 4), suggesting that apoptosis is one of major mechanisms that attribute to the low cell viability upon treatments. The apoptosis rates in SDT groups are significantly increased compared with either HMME or ultrasound treatment alone in both Ishikawa cells (p<0.05, Fig 4B) and HEC-1-a cells (p<0.01, Fig 4D). Nevertheless, SDT mainly promotes early apoptosis in Ishikawa cells (Fig 4A), while later apoptosis in HEC-1-a case (Fig 4C).

**Fig 5:**
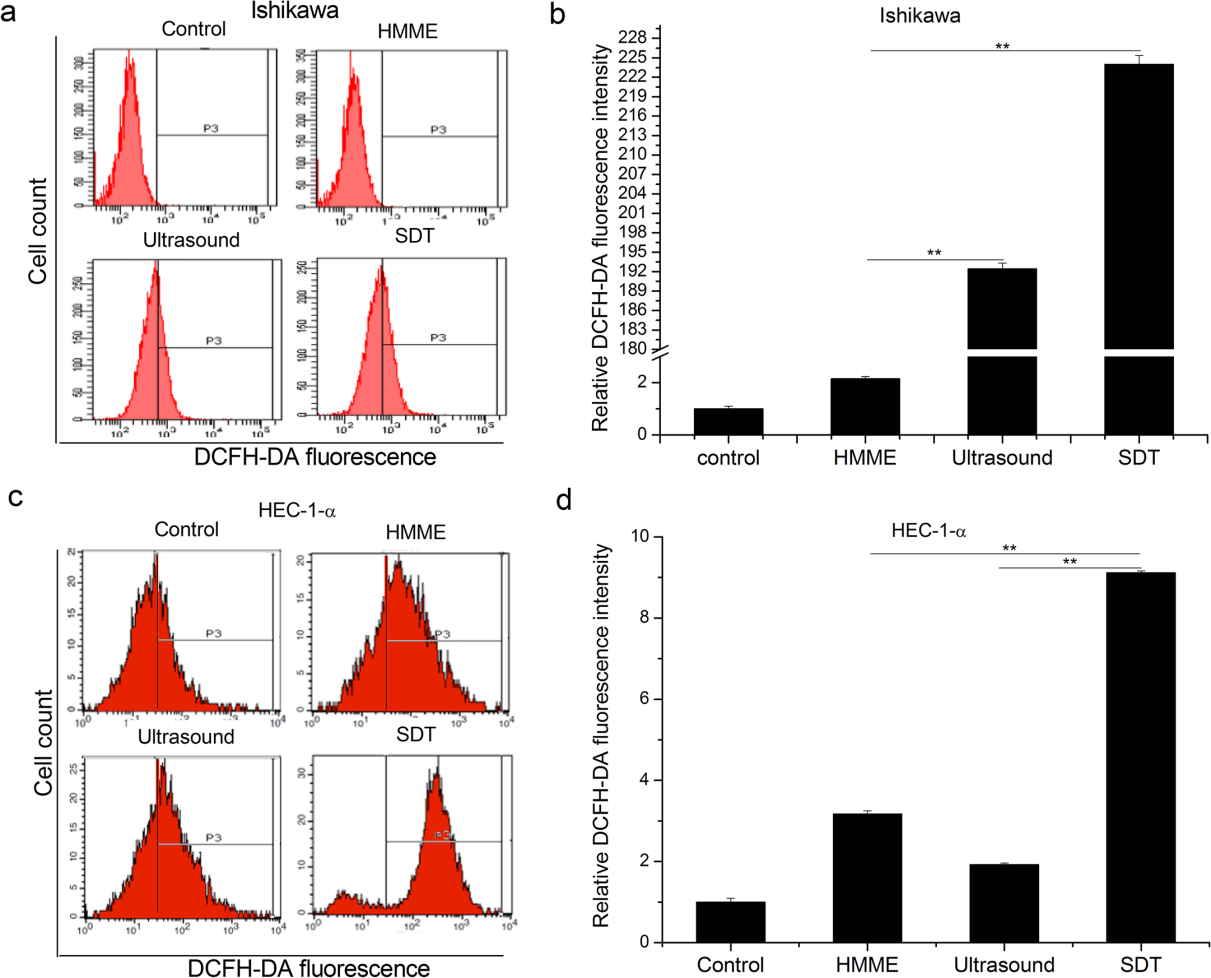
Effect of SDT on the ROS generation in endometrial cancer cells. ROS levels are significantly enhanced in SDT groups when compared with the control, HMME, and ultrasound groups in Ishikawa (a and b) and HEC-1-a (c and d) cells. Representative FACS profiles were shown in a and c. Histograms present mean ± SD of three independent experiments (b and d, ** P < 0.01.)

Intracellular ROS are important mediators of apoptosis [15]. To understand whether SDT induced apoptosis is due to ROS production, ROS levels was visualized by DCF-A, a probe that fluoresces when oxidized. It is found that among four different groups, SDT can most significantly increase the ROS levels in both Ishikawa and HEC-1-a cells (p<0.01, Fig 5). Compared with ultrasound, the ROS generation rate of SDT groups are increased up to 1.48-fold in Ishikawa cells (p<0.01) and 4.7-fold in HEC-1-a cells (p<0.01), respectively.

**Fig 6:**
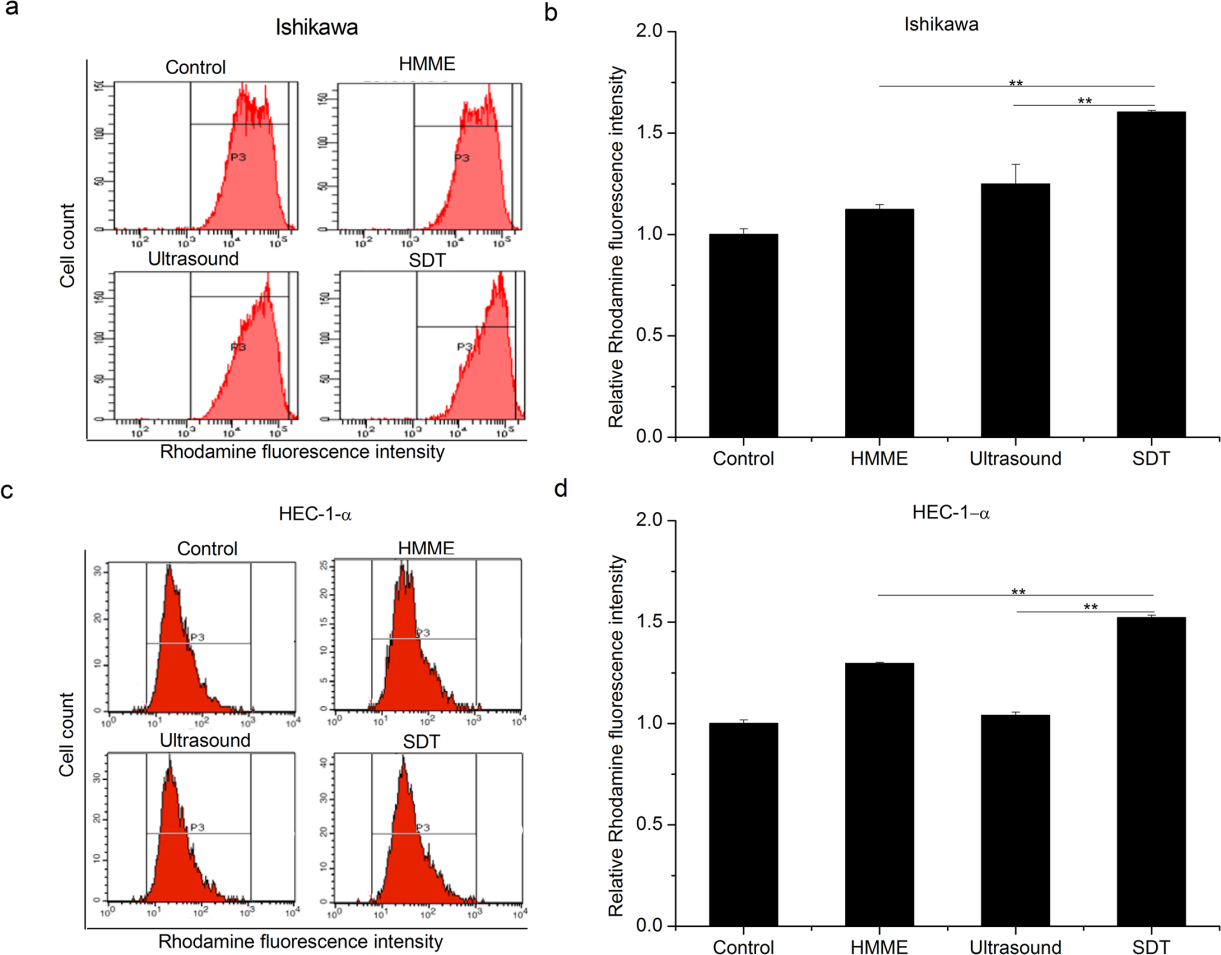
Effect of SDT on the MMP reduction in endometrial cancer cells. Ishikawa (a and b) and HEC-1-a (c and d) were subjected to four different treatments: control, HMME, ultrasound and SDT. Loss of MMP was more evident in SDT group than the other groups. Representative FACS profiles were shown in a and c. Histograms present mean ± SD of three independent experiments (b and d, ** P < 0.01.)

Enhanced ROS can induce changes of MMP, which further promotes apoptosis progression [16]. The mitochondrial membrane potential was thus measured from cells treated with Rhodamine. As shown in Fig 6, a slight decrease of MMP is observed in HMME and ultrasound groups for both Ishikawa and HEC-1-a cells. Importantly, loss of MMP becomes much more evident in SDT group compared with the mono-treatment groups in both cell lines involved. (p<0.01)

### 3.4. Effect of SDT on Intracellular Ca Ion Concentration

**Fig 7:**
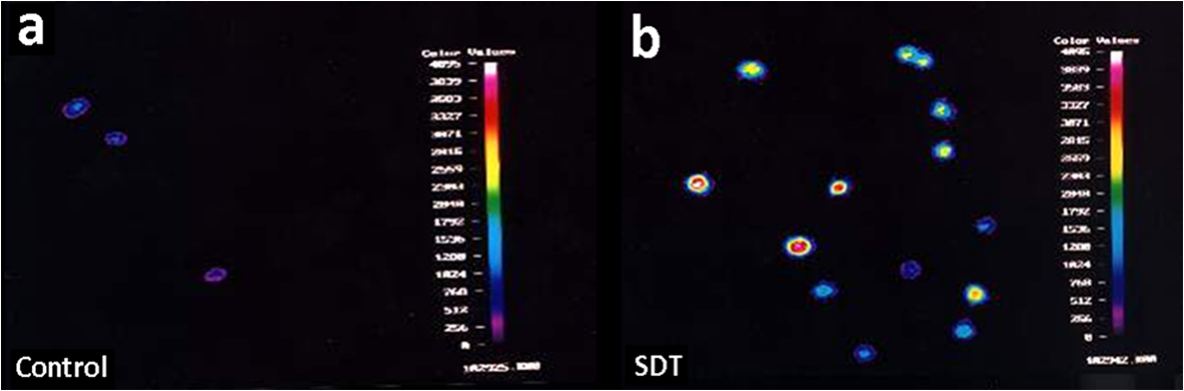
Effect of SDT on intracellular Ca ion concentration. Fluorescence intensity of Ishikawa endometrial cancer cells 1 h after the treatments was tested by a laser scanning confocal microscope (Meridian, Insight-PlusIQ) at an argon laser excitation of 488 nm. (a) Control and (b) SDT group, showing the intracellular calcium ion concentration in SDT is much higher than that in Control group.

As indicated in Fig 7 and Table 1, the Ca ion concentration in Ishikawa endometrial cells for the control, HMME, ultrasound and SDT group is of 92.533 nmol, 204.539 nmol, 2991.485 nmol and 3455.750 nmol respectively. Apparently, intracellular Ca^2+^ concentration by ultrasound treatment can be increased to 32.3 times in comparison to that of the control group, and SDT can induce a further increase of the concentration into about 37.3 times.

**Table 1:** The concentration of Ca^2+^ (nmol/l) in Ishikawa endometrial cancer cells.

Group	N	Minimum value	Maximum value	$\bar{x}\pm \text{s}$
**Control**	10	74.498	115.592	92.533 ±12.868
**HMME**	10	152.289	293.428	204.539±46.172
**Ultrasound**	10	1352.247	5131.403	2991.485±1116.140
SDT*	10	1466.053	7542.348	3455.750±2142.808

* p<0.05

### 3.5. Effect of SDT on the Expression of Apoptotic-Related Proteins

**Fig 8:**
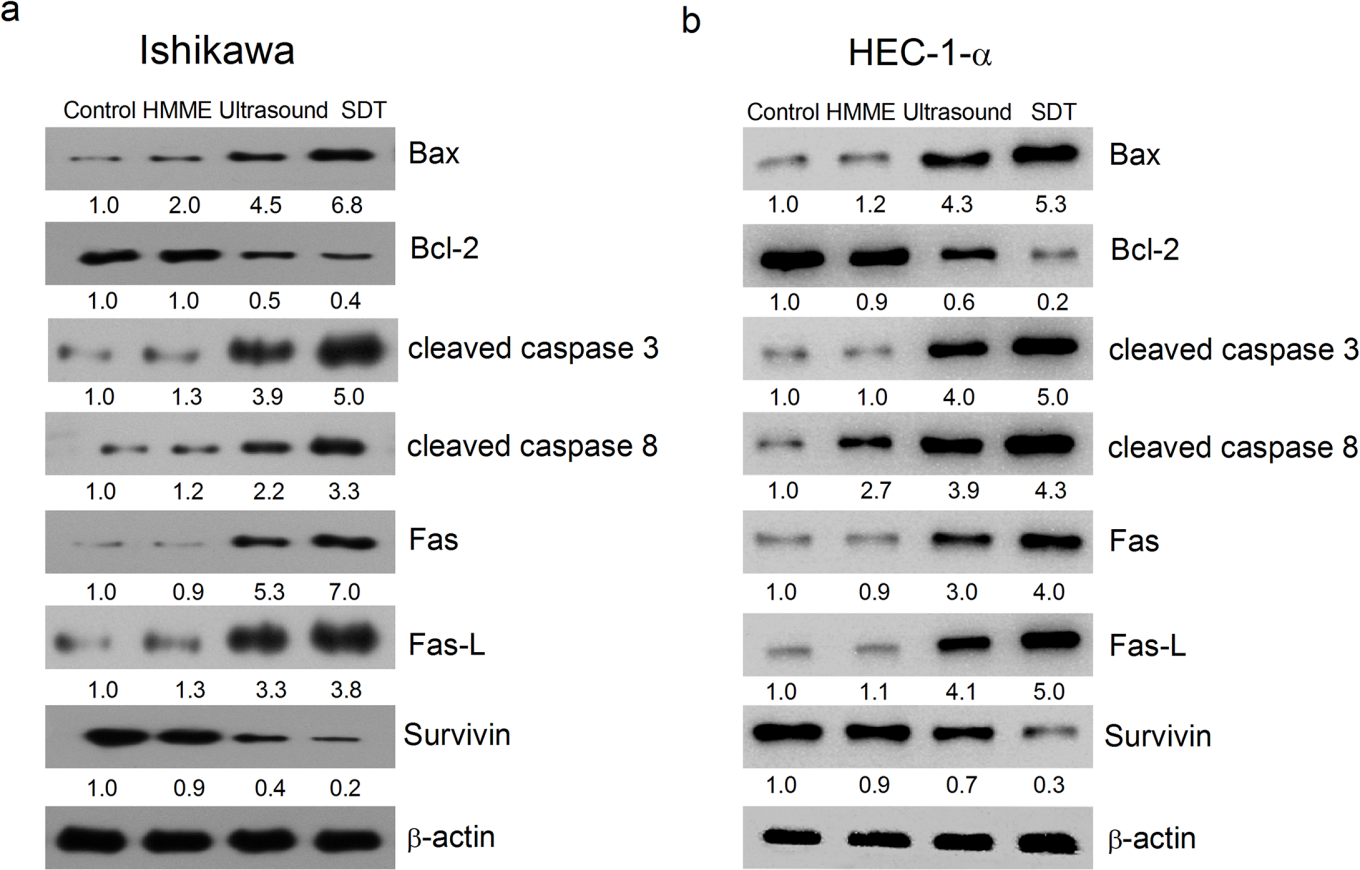
Effect of SDT on the expression of apoptotic-related proteins by western blot. Ishikawa (a) and HEC-1-a (b) were subjected to four different treatments: control, HMME, ultrasound and SDT. Apoptosis related markers were detected by Western blotting with specific antibodies as indicated. β-actin was used a loading control.

In order to understand the molecular mechanisms of SDT-induced apoptosis, the activation of caspase-3 and caspase-8 was first detected by western blotting after different treatments. As shown in Fig 8, both cleaved caspase-3 and caspase-8 are significantly enhanced in SDT groups, suggesting both casapses are activated upon the treatment. In addition, the anti-apoptotic protein survivin and Bcl-2 are decreased and the pro-apoptotic Bax is increased with SDT. Furthermore, death receptor pathway markers, such as Fas and Fas-L, are also enhanced by SDT (Fig 8). These data together suggested that both intrinsic and extrinsic pathways have been involved in SDT-induced apoptosis.

## Discussion

Although ultrasound has been considered as an effective treatment for cancers, unsatisfactory killing effects have been suggested by HEC-1-a cells in this study and also by previous work [7]. Importantly, SDT not only acts efficiently on ultrasounds sensitive Ishikawa cells, and also the ultrasounds resistant HEC-1-a cells, indicating that SDT may have applications on wider range of cancer cells. The synergistic pro-apoptosis effect by the combination of HMME and ultrasound in endometrial cancers may be attributed to two factors, i.e. the high efficiency of HMME as a sonosensitizer and the killing effect of ultrasound.

SDT normally has two killing modes on tumor cells, i.e. necrosis and apoptosis [17]. The dominant apoptotic-like change of cell morphology and substructure shown in the inverted microscope and TEM images points out that apoptosis is the major death manner of endometrial cancer cells in the present case. Based on our experimental results, possible mechanisms of SDT on apoptosis of endometrial cancer cells are proposed as following:

(1) Loss of MMP by ROS generation and Bcl-2 family alternation may be one of SDT-induced apoptosis pathways.

It is revealed that ROS in cells treated by SDT is most excessive among all samples. Under normal conditions, the cells themselves can delete continuously small molecules generated during aerobic respiration, including singlet oxygen [18,19]. However, at an imbalanced state, intracellular ROS can be accumulated, damage mitochondrial membrane and reduce the membrane potential [20–22]. This is consistent with the experimental results, where the most significant ROS generation rate and MMP reduction rate were observed simultaneously in SDT groups. In addition, mitochondrial membrane potential is also subjected to Bcl-2 family members’ regulation. In this study, Bcl-2 family member Bax is increased and Bcl-2 is decreased in SDT groups. The increased Bax may thus form Bax/Bax homodimers or antagonizes Bcl-2’s anti-apoptosis effect through forming Bax/Bcl-2 heterodimers at outer membrane of mitochondria [23]. Such changes can then increase membrane permeability so that apoptosis-promoting factors, such as cytochrome c, will release from mitochondria to the cytosol, where they trigger caspase cascades and eventually lead to apoptosis of endometrial cancer cells. Therefore, we conclude that excessive ROS and changes of Bcl-2 family members in SDT may activate the intrinsic apoptosis pathway by affecting mitochondria membrane potentials.

(2) Overloaded Ca ions in cells may be another major intrinsic factor for the cell apoptosis in SDT.

The overload of Ca ions involves various apoptosis processes, in particular the upstream regulation ones. Ca ions are normally stored in endoplasmic reticulum. The endoplasmic reticulum stress due to excessive ROS [14] can increase Ca^2+^ storage capacity of cells as well as the release speed of Ca^2+^ into the cells. As demonstrated by the present confocal microscope results, Ca^2+^ overload in cells is rather severe for SDT group. Since the release of cytochrome c has a positive feedback on the Ca^2+^ concentration [24–26], the Ca^2+^ overload in SDT can be another stimulant for the large release of cytochrome c, which then activates Caspase-3 and induce cell apoptosis as mentioned above.

(3) SDT may effectively silence surviving gene.

Recently, it has been demonstrated that the survivin expression rate in endometrial carcinoma tissue is (100%) higher than that in longer endometrial hyperplasia (73%) [27], implying that survivin may be closely associated to malignant transformation of endometrium. More importantly, earlier work has pointed out that the survival protein survivin can strongly inhibit apoptosis stimulated by diverse factors [28]. Silencing survivin may therefore break the protection and favor the apoptosis of cancer cells. In this work, SDT has been demonstrated to be an effective way to suppress this gene, leading to an extraordinarily weakened expression of survivin and more effective activation of caspase-8 and caspase-3 compared with the three other groups.

(4) Extrinsic pathway may assist the intrinsic factors for apoptosis in SDT.

The presence of Fas/Fas-l is a sign of the death receptor-mediated extrinsic apoptosis pathway where caspase-8 acts as an important initiation caspase and caspase-3 is the executive caspase. Our results show that the death receptor proteins Fas/Fas-l significantly increased in SDT groups and both caspase-8 and caspase-3 are activated with the same treatment, which directly indicates the activation of extrinsic apoptotic pathways in response to SDT.

In summary, SDT can significantly inhibit the proliferation of endometrial cancer cells, much more effective than HMME or ultrasound alone in both ultrasound sensitive and resistant cells. The apoptosis induced by HMME-SDT involves multiple pathways, including intrinsic apoptotic pathway activated by ROS generation, MMP reduction and Ca^2+^ overload, effective silence of survivin gene, together with Fas/Fas-l-mediated extrinsic apoptosis pathway. Considering the remarkable effectivity in both ultrasound sensitive and resistant cells, HMME-SDT may therefore have opened up a new rehabilitation road for patients who cannot survive from surgery, radiotherapy or chemotherapy, and who require preserving organs and even reproductive functions.

## Supporting Information

S1 Fig Ultrasound sensitivity of endometrial cancer cells analyzed by CKK-8 assays. (a) Ishikawa and HEC-1-a cells were treated with ultrasound (1 MHz) at the intensity of 1.0 W/cm^2^ for 60 s and then subjected to a CKK-8 assay. Ishikawa is more sensitive to ultrasound treatment than HEC-1-a. Data are presented as the mean ± SD (n = 3), **P < 0.01. (b) Ultrasound resistant HEC-1-a cells were treated with ultrasound at an increased intensity of 2.0 W/cm^2^ for 0 s, 60 s, 120 s, and 240 s, respectively. A slight and time-dependent cell viability inhibition is observed with the treatments. Data are presented as the mean ± SD (n = 3).
(TIF)
