# Supplementary figures and images for: Apoptosis-Promoting Effects of Hematoporphyrin Monomethyl Ether-Sonodynamic Therapy (HMME-SDT) on Endometrial Cancer
Source: PLoS One. 2015 Sep 14;10(9):e0137980. doi: 10.1371/journal.pone.0137980 (PMC4569302; doi:10.1371/journal.pone.0137980)

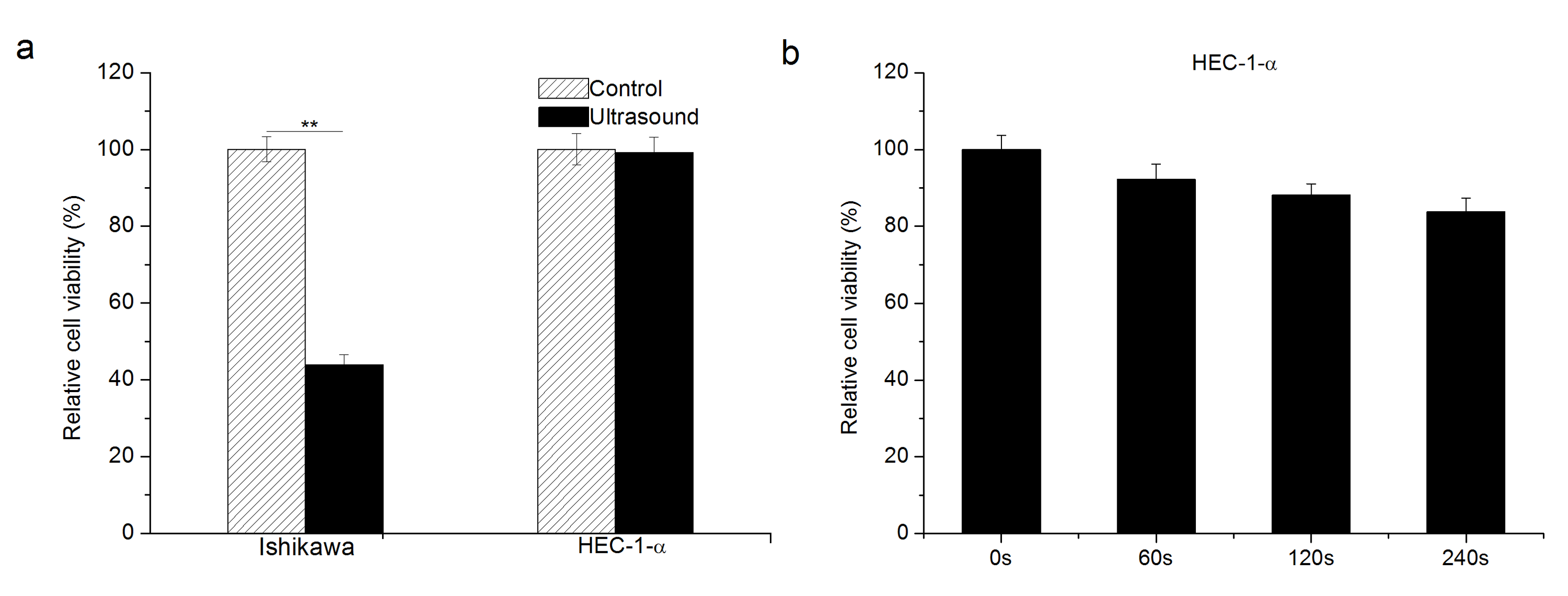

Supplement: S1 Fig — (a) Ishikawa and HEC-1-a cells were treated with ultrasound (1 MHz) at the intensity of 1.0 W/cm2 for 60 s and then subjected to a CKK-8 assay. Ishikawa is more sensitive to ultrasound treatment than HEC-1-a. Data are presented as the mean ± SD (n = 3), **P < 0.01. (b) Ultrasound resistant HEC-1-a cells were treated with ultrasound at an increased intensity of 2.0 W/cm2 for 0 s, 60 s, 120 s, and 240 s, respectively. A slight and time-dependent cell viability inhibition is observed with the treatments. Data are presented as the mean ± SD (n = 3). (TIF) [file pone.0137980.s001.tif]
